# Inter-rater reliability of the Full Outline of UnResponsiveness score and the Glasgow Coma Scale in critically ill patients: a prospective observational study

**DOI:** 10.1186/cc8963

**Published:** 2010-04-14

**Authors:** Michael Fischer, Stephan Rüegg, Adam Czaplinski, Monika Strohmeier, Angelika Lehmann, Franziska Tschan, Patrick R Hunziker, Stephan C Marsch

**Affiliations:** 1 Department of Medical Intensive Care, University Hospital, Spitalstrasse, Basel, 4031, Switzerland; 2 Department of Neurology, University Hospital, Spitalstrasse, Basel, 4031, Switzerland; 3 Department of Psychology, University of Neuchatel, Rue de la Maladière, Neuchatel, 2000, Switzerland

## Abstract

**Introduction:**

The Glasgow Coma Scale (GCS) is the most widely used scoring system for comatose patients in intensive care. Limitations of the GCS include the impossibility to assess the verbal score in intubated or aphasic patients, and an inconsistent inter-rater reliability. The FOUR (Full Outline of UnResponsiveness) score, a new coma scale not reliant on verbal response, was recently proposed. The aim of the present study was to compare the inter-rater reliability of the GCS and the FOUR score among unselected patients in general critical care. A further aim was to compare the inter-rater reliability of neurologists with that of intensive care unit (ICU) staff.

**Methods:**

In this prospective observational study, scoring of GCS and FOUR score was performed by neurologists and ICU staff on 267 consecutive patients admitted to intensive care.

**Results:**

In a total of 437 pair wise ratings the exact inter-rater agreement for the GCS was 71%, and for the FOUR score 82% (*P* = 0.0016); the inter-rater agreement within a range of ± 1 score point for the GCS was 90%, and for the FOUR score 92% (*P* = ns.). The exact inter-rater agreement among neurologists was superior to that among ICU staff for the FOUR score (87% vs. 79%, *P* = 0.04) but not for the GCS (73% vs. 73%). Neurologists and ICU staff did not significantly differ in the inter-rater agreement within a range of ± 1 score point for both GCS (88% vs. 93%) and the FOUR score (91% vs. 88%).

**Conclusions:**

The FOUR score performed better than the GCS for exact inter-rater agreement, but not for the clinically more relevant agreement within the range of ± 1 score point. Though neurologists outperformed ICU staff with regard to exact inter-rater agreement, the inter-rater agreement of ICU staff within the clinically more relevant range of ± 1 score point equalled that of the neurologists. The small advantage in inter-rater reliability of the FOUR score is most likely insufficient to replace the GCS, a score with a long tradition in intensive care.

## Introduction

The assessment of comatose patients is an important part of critical care. Unfortunately, there is no objective measure of coma like temperature or blood pressure. Thus, so far the assessment of the level of coma has to rely on clinical scores. The Glasgow Coma Scale (GCS), originally designed for patients with head trauma [1], has become the most widely used scoring system for patients with an altered level of consciousness in the ICU. Important limitations of the GCS include inconsistent inter-observer reliability [2], concerns over the predictive value in brain injury patients undergoing modern neuro-intensive care [3], the impossibility of assessing the verbal score in intubated patients, and the exclusion of brainstem reflexes. Over the past decades, a variety of alternative scoring systems have been developed [4-7], although none of them reached widespread acceptance.

The FOUR (Full Outline of UnResponsiveness) score, a coma scale consisting of four components (eye response, motor response, brainstem reflexes, and respiration pattern) was recently proposed by investigators from the Mayo Clinic [8]. Validation among patients receiving no sedative agents by dedicated staff in neuro-intensive care demonstrated good to excellent inter-rater reliability [8,9]. By contrast to the GCS, the FOUR score does not rely on a verbal response. In the ICU, a variety of conditions such as intubation, sedation, or delirium preclude a reliable assessment of a verbal response and, therefore, the FOUR score is an attractive tool. However, before this new score can be recommended for routine use in the ICU, the following limitations should be addressed: so far the FOUR score has not been validated in critically ill patients outside of the Mayo Clinic; so far the FOUR score has not been validated in sedated patients; and so far the FOUR score has only been validated by dedicated staff in neuro-ICUs. This may have resulted in a much higher inter-rater reliability than that achievable by ICU staff of general ICUs.

Accordingly, the aims of the present study were: to compare the inter-rater reliability of the GCS and the new FOUR score among unselected patients in general medical ICU; and to compare the inter-rater reliability of neurological scoring provided by staff members of general medical ICUs with that of neurologists.

## Materials and methods

The study was performed on one of the two subunits of the medical ICU of the University Hospital of Basel, Switzerland. The study was approved by the regional ethical committee. As GCS scoring was already routinely performed on our unit prior to the study and no therapeutic decisions were based on the FOUR scoring, the ethical committee waived the need to obtain individual informed consent.

Ratings were performed by two board-certified staff neurologists (S.R. and A.C.) serving as gold standard, eight ICU nurses, and four ICU physicians. Prior to the study, all raters received an instruction by one of the neurologists including a supervised scoring of GCS and FOUR score in two patients.

We prospectively studied the FOUR score and the GCS in consecutive adult patients admitted to our ICU. Exclusion criteria were the unavailability of both neurologists and the patients' unwillingness to participate in the ratings. Scoring was performed between 9:00 am and 10:00 am on weekdays only. Scoring occurred at the first possible occasion after admittance and each patient was scored only once. Eligible patients were identified by the head-nurse and colour-coded on the main board showing all patients presently admitted. If available, raters performed their ratings on the coded patients in the time frame specified. Raters were not aware of other ratings or the results thereof. Patients were included if at least one of the neurologists and one member of the ICU staff were able to perform a rating within a time interval of one hour. In addition, 100 consecutive patients were rated by both of the two neurologists to assess their inter-rater agreement. Patients were included if both neurologists were able to perform their ratings within a time interval of one hour.

For GCS scoring, the raters used a one-sided A4-sized form containing written instructions. In intubated patients, the rating for the verbal domain of the GCS was defined to be 1. For the FOUR scoring, the raters used a one-sided A4-sized form containing both written and visual instruction: the written instruction was a German translation of the original instruction from the Mayo Clinic [8]; the visual instruction was a coloured copy of the version published in 2005 [8], adapted in size to fit the scoring form. The definition of the FOUR score and the GCS are displayed in Table 1.

**Table 1:** Definition of the FOUR score and the Glascow Coma Score

FOUR score	Glascow Coma Scale
Eye response	Eye response
4 = eyelids open or opened, tracking, or blinking to command	4 = eyes open spontaneously
3 = eyelids open but not tracking	3 = eye opening to verbal command
2 = eyelids closed but open to loud voice	2 = eye opening to pain
1 = eyelids closed but open to pain	1 = no eye opening
0 = eyelids remain closed with pain	Motor response
Motor response	6 = obeys commands
4 = thumbs-up, fist, or peace sign	5 = localising pain
3 = localising to pain	4 = withdrawal from pain
2 = flexion response to pain	3 = flexion response to pain
1 = extension response to pain	2 = extension response to pain
0 = no response to pain or generalised myoclonus status	1 = no motor response
Brainstem reflexes	Verbal response
4 = pupil and corneal reflexes present	5 = oriented
3 = one pupil wide and fixed	4 = confused
2 = pupil or corneal reflexes absent	3 = inappropriate words
1 = pupil and corneal reflexes absent	2 = incomprehensible sounds
0 = absent pupil, corneal, and cough reflex	1 = no verbal response
Respiration	
4 = not intubated, regular breathing pattern	
3 = not intubated, Cheyne-Stokes breathing pattern	
2 = not intubated, irregular breathing	
1 = breathes above ventilator rate	
0 = breathes at ventilator rate or apnoea	

FOUR score = Full Outline of UnResponsiveness.

Acute physiology and chronic health evaluation (APACHE) II scores were obtained for the first 24 hours after admittance to the ICU. For patients that stayed for 28 days or more in our hospital or died during their hospitalisation, 28-day mortality was assessed using the in-hospital electronic patient documentation system. Twenty eight-day mortality of discharged patients was assessed by contacting the physician treating the patient at home or in another institution.

### Statistics

Data were analysed using SPSS (version 15.0), a commercially available statistical software. Three categories of pair-wise ratings were analysed: 1) neurologist - neurologist, 2) ICU staff ICU staff, and, 3) neurologist ICU staff. For each category no more than one pair-wise rating was analysed in every patient. In case of more than one pair-wise rating in a given category (e.g. patient was rated by two neurologists and two members of ICU staff resulting in four pair-wise ratings in the category neurologist ICU staff) the rating to be analysed was randomly chosen using computer-generated numbers. Pair-wise-weighted kappa values were calculated for the GCS and the FOUR score. A kappa value of 0.4 or less is considered poor, values between 0.4 and 0.6 are considered fair to moderate, values between 0.6 and 0.8 are considered good, and values above 0.8 are considered excellent agreement [10]. Although assessment of inter-rater reliability using kappa statistics is scientifically appropriate, this approach does not result in measures of obvious clinical usefulness. Rather than an exact agreement we determined that for the dynamic environment of the ICU a precision in scoring within the range of ± 1 score points for both GCS and FOUR score would be sufficient for the majority, if not all, clinical decisions based on the scoring result. Thus, an inter-rater agreement within a range of ± 1 score points for both GCS and FOUR score was chosen as primary outcome. Secondary outcomes were exact inter-rater agreements and ratings of the sub-components of the two scores. For the primary outcome, a difference of 10% or more between the agreement rates of the neurologists and of the ICU staff was considered to be of clinical relevance. We estimated that scoring around 250 patients would allow to detect that difference with an α of 0.05 and a power of 90. Anticipating a drop-out rate of around 20% we planned to include 300 patients. We decided to analyse three pre-defined sub-groups for the primary endpoint: intubated patients, sedated patients, and patients with neurological diseases as primary admittance diagnosis. As previous work reported that the motor component of the GCS (GCS-mot) has a similar predictive value as the total GCS [5], and the combined eye and motor component of the FOUR score (FOUR-EM) has a similar predictive value as the total FOUR score [11] we separately analysed the predictive values for mortality and agreement rates for the GCS-mot and the FOUR-EM. Cronbach's α [12] was calculated to assess the internal consistency of both scores. Predictive values of the scores were assessed by calculating the area under the curve (AUC) with 95% confidence intervals from receiver operating characteristic (ROC) curves. Frequency tables were analysed using Fisher's exact test. A P less than 0.05 was considered to represent statistical significance.

## Results

### Patients

The study took place between May 2006 and April 2007. During the study period 992 patients were admitted to the subunit of our ICU where the study took place. In 664 cases, patients had to be excluded because no neurologist was available or patients were unwilling to participate. Scoring was performed on 328 patients. Of the 328, 61 (33 female; mean age 62 ± 17 years; APACHE II 13 ± 7) had to be excluded because no pair-wise rating occurred within a time interval of one hour. Thus, 267 patients (85 female; mean age 63 ± 17 years; APACHE II 14 ± 8) were included in the study resulting in 437 pair-wise ratings. Pair-wise ratings of the two neurologists were obtained in 100 of the 267 patients (40 female; mean age 64 ± 16 years; APACHE II 15 ± 7). The admittance diagnoses of the 267 included patients are displayed in Table 2. At the time of scoring 60 of 267 (22.5%) patients were intubated or had a tracheostoma and 52 of 267 (19.5%) received sedative drugs in the eight hours preceding scoring.

**Table 2:** Primary admittance diagnoses of 267 patients undergoing scoring of GCS and FOUR in intensive care

Reason for admission	N
Neurologic disorders	86
Cardiac disorders	74
Pulmonary disorders	33
Infectious diseases	33
Gastrointestinal disorders	15
Metabolic and endocrinologic disorders	7
Renal disease	1
Other	18

FOUR score = Full Outline of UnResponsiveness; GCS, Glasgow Coma Scale.

### GCS vs. FOUR score

**Figure 1:**
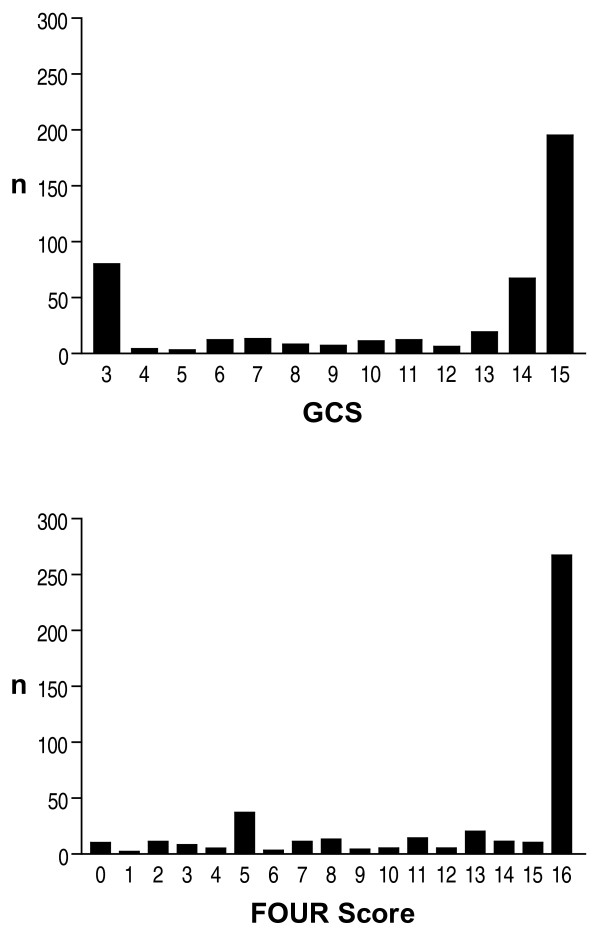
**Frequency distribution of GCS and FOUR scores**. Frequency distribution of 814 rated Glasgow Coma Scale (GCS) scores (top panel) and 814 rated Full Outline of UnResponsiveness (FOUR) scores (bottom panel).

**Figure 2:**
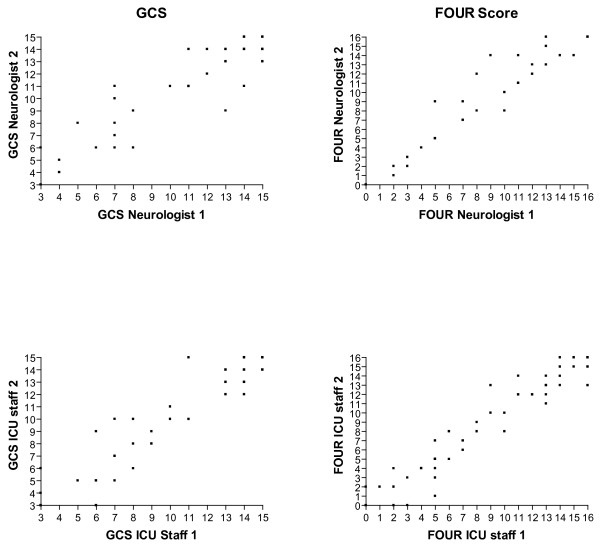
**Inter-rater agreement of GCS and FOUR scores**. Scatterplots of the pair-wise ratings of neurologists (top panels), ICU staff (middle panels), and neurologist-ICU staff (bottom panels) for the Glasgow Coma Scale (GCS; left side panels) and Full Outline of UnResponsiveness (FOUR) score (right side panels).

**Figure 3:**
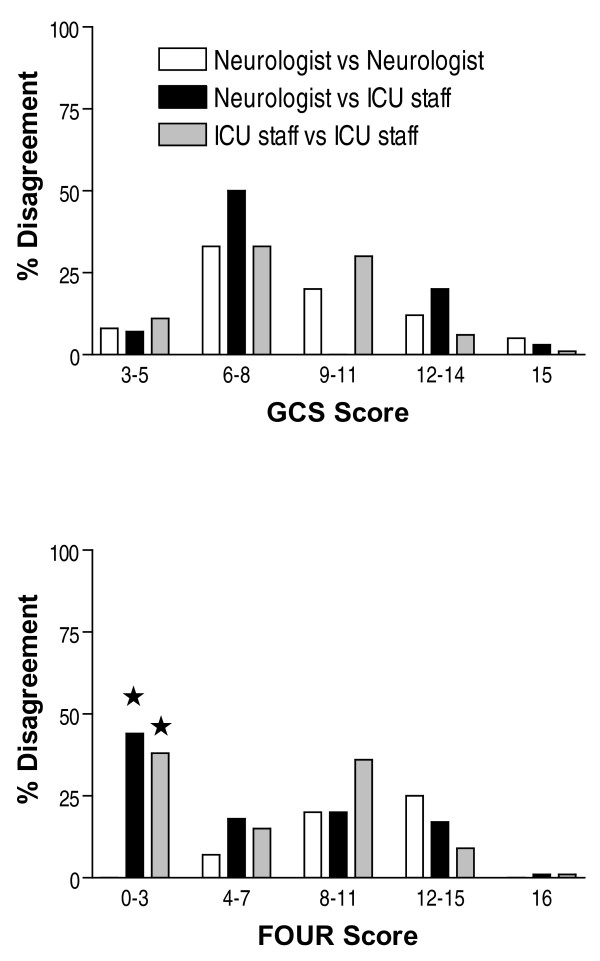
**Disagreement rates for GCS and FOUR scores**. Disagreements of more than one score point in pair-wise ratings of the Glasgow Coma Scale (GCS) score (top panel) and the Full Outline of UnResponsiveness (FOUR) score (bottom panel) respectively. Scores are divided into quartiles. As a substantial proportion of ratings were at the maximum of the each scale (i.e. GCS 15, FOUR 16), the maximum category is shown separately in addition to the quartiles. Disagreements are expressed as a percentage of the total number of ratings in a given quartile of the GCS score and FOUR score, respectively. White bars = disagreements between the neurologists; black bars = disagreements between the neurologists and ICU staff; grey bars = disagreements between ICU staff. For both scores, disagreements were significantly (*P *< 0.001) less frequent in the maximum category (i.e. GCS 15, FOUR 16) than in all other categories. * For the lowest quartile of the FOUR score, the disagreement between neurologist and ICU staff (*P *= 0.034) and between ICU staff and ICU staff (*P *= 0.045) was significantly greater than that between the neurologists.

Overall 437 pair-wise ratings were analysed. Cronbach's α for the GCS (0.87) and the FOUR score (0.83) indicate a high degree of internal consistency for both scores. The frequency distribution of the GCS and FOUR scores are displayed in Figure 1. The agreement of the ratings in the three categories is displayed in Figure 2. Overall, there was a statistically significant difference (*P *= 0.0016) with regard to exact agreement between the GCS score (71%) and the FOUR score (82%) but not for the agreement within a range of ± 1 score point (GCS 90%; FOUR 92%). Tables 3 and 4 display the kappa values for the GCS and FOUR score, respectively. Note that the inter-rater agreement of the neurologists was significantly better than that of the ICU staff with regard to the FOUR score (Table 4) but not for the GCS (Table 3). No significant difference in inter-rater agreement was found for the three components of the GCS (Table 3). In the FOUR score, however, the inter-rater agreement significantly differed between the four components with the component 'respiration' achieving the highest agreement rates and the component 'brainstem' achieving the lowest agreement rates. In addition, the agreement between the neurologists for the components 'brainstem' and 'respiration' was significantly better than that between ICU staff (Table 4). Figure 3 displays the disagreement in pair-wise ratings for both scores. As a high proportion of scorings yielded maximum scores (Figure 1) and the agreement rates were highest at theses scores (Figure 3) we calculated kappa values after excluding the maximum scores (i.e. GCS 15 and FOUR score 16, respectively) and found a significant difference between the kappas with or without excluding the maximum scores for the GCS (kappa ± 95% confidence interval, 0.61 ± 0.05 vs. 0.48 ± 0.06) and the FOUR score (0.68 ± 0.05 vs. 0.54 ± 0.08).

**Table 3:** Weighted kappa values for the interrater agreement for the GCS

Rater pair	n	Total GCS	Eye response	Motor response	Verbal response
Neurologist-Neurologist	100	0.67 ± 0.10	0.75 ± 0.12	0.79 ± 0.10	0.78 ± 0.10
Neurologist-ICU staff	393	0.56 ± 0.09	0.68 ± 0.10	0.68 ± 0.10	0.70 ± 0.09
ICU staff- ICU staff	321	0.63 ± 0.08	0.74 ± 0.09	0.78 ± 0.09	0.86 ± 0.07
Overall	437	0.61 ± 0.05	0.72 ± 0.06	0.74 ± 0.06	0.78 ± 0.05

Data = weighted kappa ± 95% confidence interval. No statistically significant differences exist between rater pairs or the different components of the GCS.

GCS, Glasgow Coma Scale.

**Table 4:** Weighted kappa values for the interrater agreement for the FOUR score

Rater Pair	n	Total	Eye response	Motor response	Brainstem reflexes	Respiration
Neurologist-Neurologist	100	0.80 ± 0.09	0.85 ± 0.09	0.88 ± 0.09	0.87 ± 0.12	1.0 ± 0.00^†^
Neurologist-ICU staff	393	0.66 ± 0.09	0.77 ± 0.09	0.73 ± 0.09	0.71 ± 0.18	0.87 ± 0.08
ICU staff- ICU staff	321	0.63 ± 0.08*	0.85 ± 0.07	0.77 ± 0.09	0.53 ± 0.16*^†^	0.87 ± 0.08*
Overall	437	0.68 ± 0.05*	0.82 ± 0.05	0.78 ± 0.05	0.67 ± 0.10	0.90 ± 0.04

Data = weighted kappa ± 95% confidence interval. * *P *< 0.05 vs. neurologist-neurologist at same component of the score; † *P *< 0.05 vs. all other components of the FOUR score with the same raters.

FOUR score = Full Outline of UnResponsiveness.

### Agreement between the neurologists

The two neurologists agreed exactly in 73% of the GCS scores and in 87% of the FOUR scores (*P *= 0.014). An agreement between the neurologists in the range of ± 1 point was observed for 88% of the GCS and for 91% of the FOUR scores, respectively (*P *= not significant (ns)). Cronbach's α showed a high internal consistency of the neurologists' ratings for both the GCS (α = 0.93) and the FOUR score (α = 0.88)

### Agreement between neurologists and ICU staff

In 163 pair-wise ratings, ICU staff agreed exactly with the neurologist in 68% of the GCS scores (*P *= ns vs. agreement of the neurologists) and in 81% of the FOUR scores (*P *= 0.011 vs. GCS; *P *= 0.14 vs. agreement of neurologists). An agreement between the ICU staff and the neurologist in the range of ± 1 point was observed for 88% of the GCS and for 91% of the FOUR scores, respectively (*P *= ns for GCS vs. FOUR; *P *= ns vs. agreement of the neurologists).

### Agreement between ICU staff

In 174 pair-wise ratings, ICU staff agreed exactly in 73% of the GCS scores (*P *= ns vs. agreement of the neurologists) and in 79% of the FOUR scores (*P *= 0.017 vs. GCS; *P *= 0.04 vs. FOUR score agreement of the neurologists). An agreement between ICU staff in the range of ± 1 point was observed for 93% of the GCS and for 88% of the FOUR scores, respectively (*P *= ns for GCS vs. FOUR; *P *= ns vs. agreement of the neurologists). The internal consistency of the ICU staffs' ratings was high for both the GCS (α = 0.87) and the FOUR score (α = 0.83).

### Predictive value for 28-day mortality

**Figure 4:**
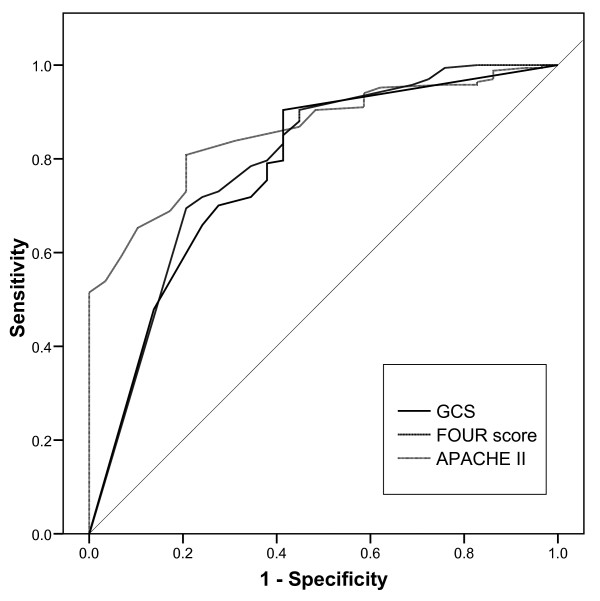
**Predictive value for 28-day mortality**. Receiver operating characteristic curve for the predictive value of Glasgow Coma Scale (GCS), Full Outline of UnResponsiveness (FOUR) score, and acute physiology and chronic health evaluation (APACHE) II score on 28-day mortality. There was no statistically significant difference between the areas under the curve of the three scores.

Twenty eight-day mortality was 13%. There was no significant difference in the predictive values of the GCS (AUC of the ROC 0.78, 95% confidence interval 0.68 to 0.87), the FOUR score (AUC of the ROC 0.79, 95% confidence interval 0.69 to 0.89), and the APACHE II score (AUC of the ROC 0.86, 95% confidence interval 0.80 to 0.92) for 28-day mortality (Figure 4). However, mortality was significantly (*P *< 0.001) higher for patients with the three lowest total FOUR scores of 0 to 2 (83% died), when compared with patients with the lowest GCS score of 3 (45% died).

### Analysis of predefined subgroups

The exact inter-rater agreement was better for the FOUR score than for the GCS in the three predefined subgroups intubated patients (n = 60; 78% vs. 65%, *P *= 0.026), sedated patients (n = 52; 73% vs. 62%, *P *= 0.095), and patients with neurological disease as primary admittance diagnosis (n = 86; 80% vs. 69%, *P *= 0.046). The exact inter-rater agreement of the neurologist and the ICU staff for the FOUR score was 79% vs. 68% for intubated patients, 88% vs. 74% for sedated patients, and 91% vs. 79% for patients with neurological disease as primary admittance diagnosis, respectively. Due to the comparatively small absolute numbers these differences failed to reach statistical significance. There was no significant difference with regard to inter-rater agreement with a range of ± 1 point between the GCS and FOUR score or different kind of rater pairs for the predefined subgroups.

The AUC of the ROC of the GCS-mot for 28-day mortality was 0.75 (95% confidence interval 0.64 to 0.86) and did not significantly differ from the total GCS or the FOUR score. Over all 437 pair-wise ratings, the exact agreement for the GCS-mot was 87% (*P *< 0.0001 vs. the total GCS; *P *= 0.006 vs. FOUR score). The agreement within a range of ± 1 score point of the GCS-mot was 95% (*P *= 0.0012 vs. GCS; *P *= 0.0002 vs. FOUR score).

The AUC of the ROC of the combined FOUR-EM for 28-day mortality was 0.76 (95% confidence interval 0.66 to 0.87) and did not significantly differ from the total FOUR score, the GCS, or GCS-mot. Overall, 437 pair-wise ratings, the exact agreement for the FOUR-EM was 85% (*P *= 0.07 vs. total FOUR score; *P *< 0.0001 vs. GCS). The agreement within a range of ± 1 score point of the FOUR-EM was 92% (*P *= 0.095 vs. total FOUR score; *P *= 0.21 vs. GCS).

## Discussion

The present study compared the inter-rater agreement of GCS and FOUR score as well as the inter-rater agreement of neurologist and ICU staff in unselected critically ill patients. In the primary outcome, i.e. the inter-rater agreement within the range of ± 1 score point, there was neither a significant difference between the GCS and the FOUR score nor a difference between neurologists and ICU staff. Exact inter-rater agreement was significantly better between neurologists than between ICU staff. Moreover, exact inter-rater agreement was significantly better for the FOUR score than for the GCS.

Recently, Wijdicks and colleagues, Wolf and colleagues, and Iyer and colleagues from the Mayo Clinic devised and validated the FOUR score [8,9,13]. Compared with the GCS, this new coma scale does not depend on a verbal response and provides greater neurological detail by inclusion of brainstem reflexes and breathing patterns. The present study is the first validation of the FOUR score in the ICU outside the institution that developed the FOUR score. In addition, the present study is the first validation of the FOUR score in unselected patients in a medical ICU. In agreement with the initial reports we observed that the inter-rater reliability for the FOUR score is at least as good as that of the GCS [8,9,13]. Moreover, our results demonstrate that the FOUR score is superior to the GCS with regard to exact inter-rater agreement. The inter-rater agreement in the present study was considerably lower for both GCS (kappa 0.59 vs. 0.82 to 0.98) and FOUR score (kappa 0.63 vs. 0.82 to 0.99) than previously reported [8,9,13]. This may be explained by the higher number of patients included in the present study, the inclusion of intubated and sedated patients, inherent variations in the level of consciousness among unselected ICU patients, organisational aspects of the scoring, and differences in the neurological expertise of the raters. Indeed, our neurologists achieved an inter-rater agreement for the FOUR score (kappa 0.80), but not for the GCS (kappa 0.67), comparable with that reported by Wijdicks and colleagues, Wolf and colleagues, and Iyer and colleagues [8,9,13]. Previous work demonstrated that the FOUR score predicts mortality as well as the GCS [8,9,11,13]. This is confirmed by our finding that the predictive value for 28-day mortality of the FOUR score equalled that of the GCS, and the APACHE II score. Moreover, in agreement with previous work our results demonstrate that mortality in medical ICU patients with the lowest FOUR score is higher than in patients with the lowest GCS.

The inter-rater agreement of the neurologists was never worse and partly significantly better than that of the ICU staff. However, as far as precision in scoring within the range of ± 1 score points is concerned, ICU staff equalled neurologists. This finding indicates that the precision in neurological scoring sufficient for the clinical settings achieved by general ICU staff cannot be significantly improved by dedicated specialists from outside the ICU.

The repetitive assessment of the level of consciousness is a routine procedure in ICU and so far the GCS is the most widely used tool. The present study confirms previous reports on a less than perfect inter-observer agreement of the GCS [2,14,15]. For the new FOUR score, the inter-rater agreement was never worse and partly better than that of the GCS. As the GCS is routinely performed in our unit, we were surprised and disappointed by the comparatively low inter-rater agreement of a longstanding standard procedure. To the best of our knowledge, there are no systematic data on the consistency of individual raters in repetitive ratings such as GCS in the ICU. Such a study would be very difficult to perform because within the time frame the level of consciousness could be kept reliably stable in critically ill patients most health-care workers would not forget their previous scoring result. It is doubtful, however, that repetitive ratings are generally more precise than the pair-wise ratings reported in the present study. Thus, our findings suggest that in the clinical setting scores of individual patients should be cautiously interpreted taking into account both the dynamic course of critical illness and inter-rater and intra-rater disagreements.

Despite its limitations, the GCS has remained the standard coma scale over the past decades. In modern ICUs, multiple scores are repetitively used. Ideally, these scores should be simple, reliable, and predictive for relevant outcomes and/or relevant clinical decisions. With regard to these criteria, the present study revealed that the FOUR score is at least equivalent and partly even superior to the GCS. Given that the inter-rater reliability of the FOUR score between the neurologists was better than that between ICU staff and that the inter-rater reliability for the brainstem component of the FOUR score was significantly lower than for the other three components there is a potential for improvement for the inter-rater reliability of the FOUR score in the settings of the ICU. By contrast, our data reveal no such potential for improvement for the GCS. Compared with the GCS, the FOUR score contains more items. In addition, the brainstem categories of the FOUR score rely on up to three different items (pupil, corneal, and cough reflex) whereas all categories of the GCS rely on one item only. Thus, the FOUR score requires more time than the GCS and is more difficult to remember in acute situations. Although the FOUR score provides more neurological detail than the GCS it cannot replace a more in-depth neurological evaluation. Balancing the advantages and disadvantages of both scores, it is fair to state that the new FOUR score is a suitable alternative to the GCS. However, we think it is unlikely that the small advantage in inter-rater reliability will prompt intensivists to replace the GCS, a score with a long tradition in the ICU, by the new FOUR score.

Eken and colleagues reported, that in patients presenting with an altered level of consciousness, head trauma, or any neurological complaints on an emergency department, the FOUR-EM had a similar predictive value for unfavourable outcomes as the total FOUR score and the GCS [11]. Their finding is in agreement with the work of Gill and colleagues showing that the three individual GCS components alone performed similar to the total GCS score for the prediction of 4 clinically relevant TBI outcomes [5]. The present study confirms and extends these previous findings by demonstrating that among unselected critically ill patients in the medical ICU the predictive values of the FOUR-EM and of the motor component of the GCS for 28-day mortality does not differ from the total FOUR score or the GCS. Moreover, the inter-rater agreements for the FOUR-EM and the GCS-mot in the present study were better than for the total FOUR score and the GCS. Thus, reducing the complexity of a score can substantially improve inter-rater reliability without necessarily losing predictive power. In a multivariable analysis in over 8,000 head trauma patients Murray and colleagues [16] found that in addition to the GCS-mot, pupil reaction has an independent predictive value. Therefore, we tested whether adding the information on bilateral pupil reactivity to the FOUR-EM would significantly increase the predictive value for 28-day mortality, which was not the case.

A limitation of our study is that due to the inclusion of unselected, and especially sedated, patients and the maximum time interval allowed for pair-wise ratings of one hour no perfectly stable experimental conditions for scoring were achieved. Particularly, we cannot exclude that some inter-rater disagreements are caused by true alterations in the level of consciousness. However, as raters performed the GCS and the FOUR score simultaneously such true alterations in the level of consciousness cannot explain the observed differences in the inter-rater agreement between the two scores. Moreover, our study conditions reflect the dynamic environment in the ICU so that our results give a fair estimate of the reliability of two coma scales in daily practice. A further limitation of our study is that no surgical patients, and especially no head trauma cases, were included so that the findings relate to unselected medical critically ill patients only. An inherent limitation of the validation of coma scales is the absence of an objective measure of the level of coma. Thus it should be kept in mind that better inter-rater reliability does not necessarily mean better accuracy.

## Conclusions

The FOUR score performs better than the GCS with regard to exact inter-rater agreement, but not for the clinically more relevant agreement within the range of ± 1 score point or the predictive value for 28-day mortality. Although neurologists outperform ICU staff with regard to exact inter-rater agreement, the inter-rater agreement of ICU staff within the clinically more relevant range of ± 1 score point equals that of the neurologists. Thus, a precision in neurological scoring sufficient for the clinical settings cannot only be achieved by dedicated staff in specialised neuro-ICUs but also by ICU staff in general ICUs. The small advantage in inter-rater reliability of the FOUR score is most likely insufficient to replace the GCS, a score with a long tradition in the ICU.

## Key messages

• The FOUR score, a new coma scale not relying on verbal response, performs better as the GCS with regard to exact inter-rater agreement, but not for the clinically more relevant agreement within the range of ± 1 score point.

• In neurological scoring, the inter-rater agreement within the range relevant for clinical decisions of ICU staff equals that of neurologists.

## Abbreviations

APACHE: acute physiology and chronic health evaluation; AUC: area under the curve; FOUR: Full Outline of UnResponsiveness; FOUR-EM: combined eye and motor component of the FOUR score; GCS: Glascow Coma Scale; GCS-mot: motor component of the GCS; ROC: receiver operator characteristics.

## Competing interests

The authors declare that they have no competing interests.

## Authors' contributions

MF participated in the design of the study, performed the neurological scoring, and was responsible for data management. SR participated in the design of the study and performed the neurological scoring. AC performed the neurological scoring. MS was responsible for identifying suitable patients and performed the neurological scoring. AL participated in the design of the study and performed the neurological scoring. FT participated in the design of the study and performed the statistical analysis. PH participated in the design of the study and performed the neurological scoring. SM participated in the design of the study, performed the neurological scoring, and drafted the manuscript. All authors contributed to the interpretation of the results and read and approved the final manuscript.
